# Rapid and Efficient Optimization Method for a Genetic Transformation System of Medicinal Plants *Erigeron breviscapus*

**DOI:** 10.3390/ijms24065611

**Published:** 2023-03-15

**Authors:** Yujun Zhao, Yifan Yu, Juan Guo, Yifeng Zhang, Luqi Huang

**Affiliations:** 1 State Key Laboratory of Dao-di Herbs, National Resource Center for Chinese Materia Medica, China Academy of Chinese Medical Sciences, Beijing 100700, China; zhaoyj@nrc.ac.cn (Y.Z.);; 2 School of Food and Biological Engineering, Jiangsu University, Zhenjiang 212013, China

**Keywords:** *Erigeron breviscapus*, medicinal plant, biolistic, transformation, regeneration, optimization strategies

## Abstract

*Erigeron breviscapus* is an important medicinal plant with high medicinal and economic value. It is currently the best natural biological drug for the treatment of obliterative cerebrovascular disease and the sequela of cerebral hemorrhage. Therefore, to solve the contradiction between supply and demand, the study of genetic transformation of *E. breviscapus* is essential for targeted breeding. However, establishing an efficient genetic transformation system is a lengthy process. In this study, we established a rapid and efficient optimized protocol for genetic transformation of *E. breviscapus* using the hybrid orthogonal method. The effect of different concentrations of selection pressure (Hygromycin B) on callus induction and the optimal pre-culture time of 7 days were demonstrated. The optimal transformation conditions were as follows: precipitant agents MgCl_2_ + PEG, target tissue distance 9 cm, helium pressure 650 psi, bombardment once, plasmid DNA concentration 1.0 μg·μL^−1^, and chamber vacuum pressure 27 mmHg. Integration of the desired genes was verified by amplifying 1.02 kb of *htp* gene from the T0 transgenic line. Genetic transformation of *E. breviscapus* was carried out by particle bombardment under the optimized conditions, and a stable transformation efficiency of 36.7% was achieved. This method will also contribute to improving the genetic transformation rate of other medicinal plants.

## 1. Introduction

*Erigeron breviscapus* (Vant.) Hand-Mazz is a perennial herbaceous plant in the Asteraceae family. This plant is distributed in Hunan, Guangxi, Guizhou, Sichuan, Yunnan, and Tibet provinces in China. It can only be seen in some open slope grasslands and forest margins at altitudes of 1200–3500 m [1] and has been listed as a nationally protected species of traditional Chinese medicine. The chemical components in *E. breviscapus* mainly include flavonoids, caffeoyls, coumarins, lignans, terpenoids, and other components [2], among which scutellarin has the highest content. Studies have shown that scutellarin can reduce neuronal damage caused by traumatic brain injury, cerebral ischemia, reperfusion injury and hypoxic-ischemic brain injury [3,4,5], and is commonly used to treat acute cerebral infarction in elderly patients [6] and is also effective in patients with diabetic nephropathy [7,8]. Another active ingredient is caffeoylquinic acids (CQAs), which can dilate blood vessels and inhibit thrombosis in vivo [9]. *E. breviscapus* is currently the best natural biological medicine for the treatment of obliterative cerebrovascular diseases and the sequela of cerebral hemorrhage, with an efficiency of >95% and slight side effects. According to the World Health Organization, the number of deaths from cardiovascular diseases will increase to 23.6 million by 2030 [10]. Therefore, *E. breviscapus* is of great significance for recovery and secondary prevention after the first onset of cardiovascular and cerebrovascular diseases. In addition, *E. breviscapus* is a natural neuroprotective agent for Alzheimer’s disease [1]. At present, according to different doses and routes of administration of *E. breviscapus* extract, various dosage forms have been developed, such as tablets, capsules, oral liquids, and injections, and international research on *E. breviscapus* unilateral preparations has also begun. It is expected to become an internationally recognized botanical medicine after ginkgo biloba preparations.

At present, scutellarin is mainly derived from extracts of *E. breviscapus*, but the cost of planting *E. breviscapus* remains high due to the limited cultivation area, low content of active ingredients, serious pests and diseases, and the degradation of germplasm variation. A yeast cell factory for the total synthesis of scutellarin has been successfully constructed in the *Saccharomyces cerevisiae* chassis cells, and the total synthesis of scutellarin has been achieved, but it is still a certain distance from industrial production [11]. Therefore, urgent measures are needed to develop varieties with high content of active ingredients, high yield, and disease resistance to achieve sustainable utilization of resources.

The development of genetic manipulation, DNA technology, and genetic transformation provides a reliable route for the development of transgenic medicinal plants that are tolerant to abiotic stress, resistant to pests, and have excellent agronomic traits [12]. Direct genetic transformation has become the method of choice for basic plant research and the primary technique for generating transgenic plants [13,14]. Therefore, the development of genetic transformation technology provides an opportunity to accelerate the various improvements of *E. breviscapus*.

The two main methods of plant genetic transformation are *Agrobacterium*-mediated transformation and biolistic-mediated transformation [15,16]. Particle bombardment is simple to perform and is virtually unlimited in terms of plant ranges and material genotypes [17]. Various types of transformation receptors are suitable for large-sized genetic cargo [18]. Target genes can be introduced into the organelles of plant cells. Therefore, the biolistic-mediated transformation is widely used in the current transgenic research. *Agrobacterium*-mediated transformation is more efficient in dicots than in monocots and is limited to specific plant host ranges [15,19,20]. On the other hand, *Agrobacterium*-mediated transformation is generally considered to be more precise and controllable than particle bombardment; however, a report on bombardment strategies [13,21] showed little evidence of major differences in levels of transgene instability and silencing when compared in the same species as in other non-model systems. Both approaches have their advantages and limitations [22,23]. At present, regenerated plants of different explants of *E. breviscapus*, i.e., true leaves [24], petioles [25], and anther [26], have been successfully obtained and plant regeneration programs have been established. He et al. established an *Agrobacterium*-mediated transformation system and applied it to the functional verification of biosynthetic pathway genes [27]. Qiu screened suitable acceptor materials for gene gun induction [28].

In this study, the leaves of *E. breviscapus* were used as explants, and different particle bombardment parameters were optimized using an orthogonal design. After range analysis and variance analysis, the main influencing factors were identified, and the experimental effects were accurately and comprehensively evaluated to obtain the optimal transformation protocol. This is a successful attempt to directly transform *E. breviscapus* by gene transfer using the particle gun, which fully demonstrates the possibility of stable transformation of *E. breviscapus*. This study has long-term significance for the genetic engineering research of medicinal plants.

## 2. Results

### 2.1. Selection Pressure

In plant genetic transformation, the principle of using antibiotics is that they can effectively inhibit the growth, development, and differentiation of non-transformed cells or plants, but do not affect the normal growth of transformed cells or plants, or have little effect on transformed cells. Hygromycin B (Hyg) is widely used in many crops as an ideal screening reagent due to its high selection efficiency and small genotype differences. The induction results of different hygromycin treatments on *E. breviscapus* callus (Table 1) showed that callus induction frequency drastically decreased as the hygromycin concentration increased. After 2 weeks of culture, only very few calluses initiated from the cut edge on 2.5 mg·L^−1^ and 5.0 mg·L^−1^ Hyg. After 4 weeks of culture on medium containing 5 mg·L^−1^ Hyg, explants turned brown and were completely necrotic. Under 7.5 mg·L^−1^ Hyg, explants did not develop calluses. Therefore, 7.5 mg·L^−1^ was the lethal dose for leaf explants. Hyg significantly delayed and inhibited callus initiation and growth as the selection concentration increased. Thus, we used stepwise increasing concentration of Hyg from 2.5 mg·L^−1^ for 2~4 weeks post-transformation, and 5.0 mg·L^−1^ for 1–2 months in the callus selection and plant regeneration.

### 2.2. Pre-Culture Period

The principle of single-variable experimentation was used to confirm the suitable pre-culture period. The results showed that pre-culture of leaf explants prior to bombardment could enhance transformation efficiency. The optimal pre-culture time was 7 d. For leaf explants, when the pre-culture time was 2–5 d, or more than 3 weeks, most of the explants decayed at the incision after particle gun bombardment, and then gradually died. When the pre-culture time exceeded 7 d, the transformation efficiency did not increase, although more resistant calluses could be produced (Table 2). Therefore, the optimal pre-culture time established in this study was 7 d.

### 2.3. Transformation Conditions

**Figure 1:**
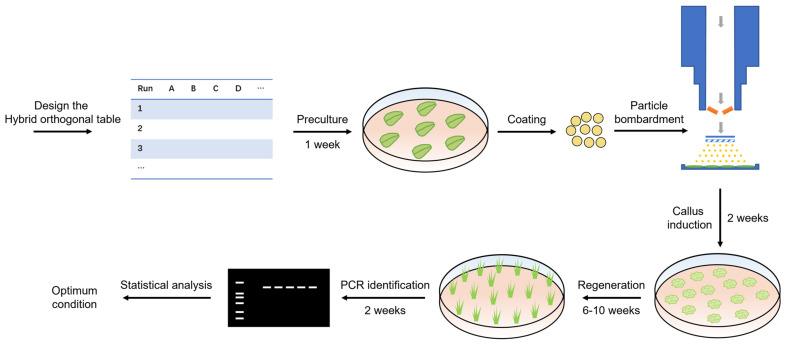
Particle-bombardment-mediated genetic transformation system optimization strategy of *E. breviscapus*.

In this study, a hybrid orthogonal experiment was adopted to confirm the optimal transformation conditions (Table 3). The particle gun transformation conditions were optimized using one two-level factor (precipitation agents) and five three-level factors (target tissue distance, helium pressure, bombardment times, plasmid DNA concentration, and chamber vacuum pressure). The experimental procedure is shown in Figure 1. The number of transformed adventitious buds was used to assessed transformation efficiency.

**Figure 2:**
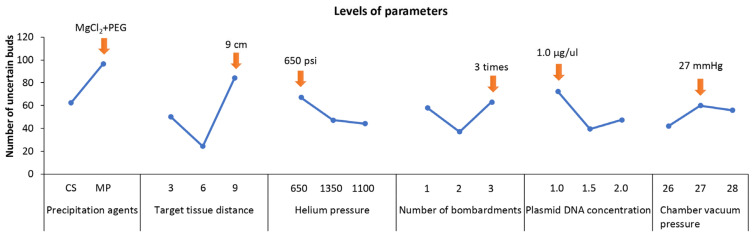
Effect of different levels of six parameters on the survival of adventitious buds after three cycles of selection.

The results showed the differences of each parameter at different levels (Figure 2). Among them, the peak of the curve showed a significant level. Specifically: precipitation agent: MgCl_2_ + PEG (level 2), target tissue distance: 9 cm (level 3), helium pressure: 650 psi (level 1), number of bombardments: 3 times (level 3), plasmid DNA concentration: 1 μg·μL^−1^ (level 3), chamber vacuum pressure: 27 mmHg (level 2).

The range value R’ is calculated by the formula (Materials and Methods 4.7). The value of R’ is used to determine the degree of influence of various factors on the conversion efficiency. The influence order of each parameter was as follows (Table 4): precipitation agents > target tissue distance > plasmid DNA concentration > number of bombardments > helium pressure > chamber vacuum pressure.

Although the results of the range analysis are more intuitive, they cannot be used to distinguish the fluctuations caused by experimental conditions or experimental errors. However, analysis of variance can be used to compensate for the shortcomings of range analysis. The significance of the parameters was determined by the *F*-test. To confirm the significant differences between the six parameters, the variance analysis was performed (Table 5). The results showed that precipitant (*p* < 0.05), target tissue distance (*p* < 0.01), and plasmid DNA concentration (*p* < 0.05) had a significant effect on transformation efficiency. However, helium pressure, number of bombardments, and chamber vacuum pressure were not significant. Therefore, the optimal transformation conditions were determined as follows: target tissue distance 9 cm, precipitant MgCl_2_ + PEG, and plasmid DNA concentration 1.0 μg·μL^−1^. The remaining transformation conditions could be considered from the perspective of economy and operation, with one bombardment, helium pressure 650 psi, and chamber vacuum pressure 27 mmHg.

### 2.4. Selection and Plant Regeneration

**Figure 3:**
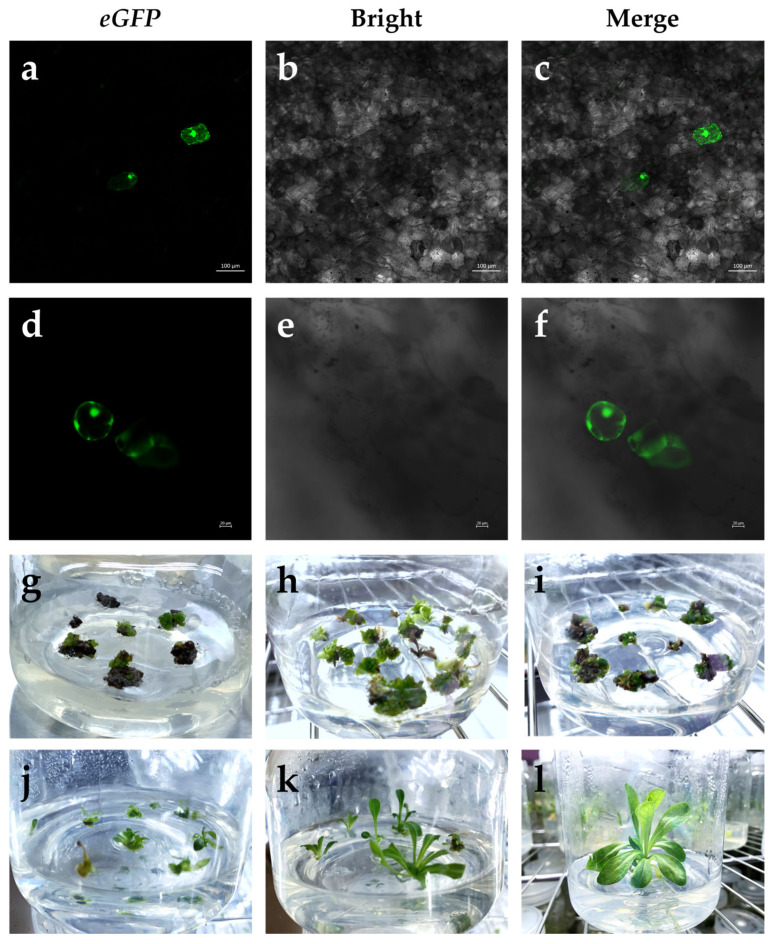
Particle-bombardment-mediated transformation, selection, regeneration, and *eGFP* fluorescent assay: (**a**–**c**) transient *eGFP* expression in leaves, scale bar 100 μm; (**d**–**f**) transient *eGFP* expression in transformed callus, scale bar 50 μm; (**g**) transformed callus under the first round (2 weeks) of selection; (**h**) transformed callus under the second round (4 weeks) of selection; (**i**) transformed callus under the third round (6 weeks) of selection; (**j**) shoot regeneration; (**k**) shoot elongation; (**l**) root formation.

Transformed adventitious shoots were randomly selected 48 h after bombardment, and transient *eGFP* expression was detected by Laser Scanning Confocal Microscope (LSCM) (Figure 3a–c). The transformants were then transferred to callus induction medium (CIM; Murashige and Skoog (MS) + 1.0 mg·L^−1^ 6-benzylaminopurine (6-BA) + 0.1 mg·L^−1^ 1-naphthylacetic acid (NAA) + 3% sucrose + 0.4% phytagel) for 2 weeks and detected by LSCM. The presence of *eGFP* in the transgenic cells was confirmed by green fluorescent spots (Figure 3d,e). After callus formation, transformants were transferred to selection medium (CIM) containing 2.5 mg·L^−1^ Hyg for the first round of selection for 2 weeks (Figure 3g). The transformants were then transferred to a regeneration medium (RM; MS + 2.0 mg·L^−1^ 6-BA + 0.2 mg·L^−1^ NAA + 3% sucrose + 0.4% phytagel, pH 5.8) containing 2.5 mg·L^−1^ Hyg for a second round of selection for 4 weeks (Figure 3h). For efficient selection, regenerated transformants were transferred to RM containing 5.0 mg·L^−1^ Hyg for a third round of selection for 6 weeks (Figure 3i). When selection was performed on RM containing Hyg, the non-transgenic tissues gradually turned brown while the presumed transformed tissues remained green and grew slowly (Figure 3g–i). The transformation efficiency was 36.7%. The transformation efficiency was calculated as the number of positive shoots that survived after the third round of selection relative to the total number of explants bombarded. After three subculture cycles, the transformed buds were transferred to a shoot elongation medium (SEM; 1/2MS + 2.0 mg·L^−1^ 6-BA + 0.2 mg·L^−1^ NAA + 3% sucrose + 0.4% phytagel, pH 5.8) for shoot elongation (Figure 3j,k). Finally, transformed rootless seedlings were transferred to a root induction medium (RIM; 1/2MS + 0.3 mg·L^−1^ NAA + 0.5 mg·L^−1^ indole-3-butyric acid (IBA) + 3% sucrose + 0.4% phytagel, pH 5.8) for further root formation for about 3 weeks (Figure 3l).

### 2.5. Identification of Transgenic E. breviscapus Plants

**Figure 4:**
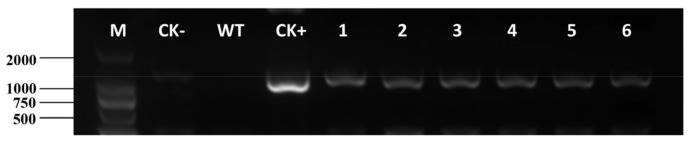
Molecular analysis of transgenic plants. PCR amplification of the *htp* gene (1026 bp). M: marker 2000; CK+: positive control (plasmid PBI-1300); CK−: negative control (H_2_O); WT: wild-type plant, lanes 1–6, putative transformants.

Putative transgenic lines were obtained by multiple rounds of selection following bombardment of *E. breviscapus* leaf explants. These putative transgenic lines were identified by PCR amplification using the specific primers (Hyg-F/R) and all the products were sequenced by Sanger sequencing to verify the *htp* gene. The results showed the expected bands of 1026 bp fragment, confirming that the *htp* gene had been integrated into the genome of *E. breviscapus*, while the wild-type (WT) plant showed no PCR amplification bands (Figure 4).

## 3. Discussion

One of the most effective methods for direct gene transfer is the particle bombardment method. Bacteria are not required during the transformation process. It is widely used and efficient in DNA transfer in mammalian cells, microbes, and monocots [29]. So far, particle bombardment has been used in many medicinal plants, i.e., *Catharanthus roseus* [30], *Hypericum perforatum* [31], *Centella asiatica* [32], *Tripterygium wilfordii* [33], *Momordica charantia* [34], *Scoparia dulcis* [35], etc. In this study, a simple and effective biolistic transformation method was established in *E. breviscapus* with a transformation efficiency of 36.7%.

The use of orthogonal design can reduce the number of experiments and the complexity of experimental analysis methods, overcome the blindness in condition optimization, and improve work efficiency and experimental accuracy. The method of biolistic transformation of *E. breviscapus* was optimized by the hybrid orthogonal design. The main influencing factors were precipitation agents, target tissue distance, and plasmid DNA concentration. The optimum transformation conditions were precipitant agents MgCl_2_ + PEG, target tissue distance 9 cm, helium pressure 650 psi, bombardment once, plasmid DNA concentration 1.0 μg·μL^−1^, and chamber vacuum pressure 27 mmHg. At present, a universal optimization strategy (Figure 1) has been developed and applied to a variety of medicinal plants, including *Erigeron breviscapus*, *Salvia miltiorrhiza*, *Tripterygium wilfordii* [33], and *Aconitum carmichaelii*. The explants used include leaves, stems, suspension cells, and calluses.

By optimizing the pre-incubation time, we found that 7–14 days of pre-cultivation was more conducive to the regeneration of explants after bombardment. However, shorter (2–5 d) or longer (21 d) pre-culture times were unfavorable to explants’ regeneration. The same conclusion was reached in the biolistic transformation of wheat-microspore-derived calluses and microspores. Pre-culture for 3–8 days could improve the GUS expression in microspores [36]. The results of optimization experiments of *Fistulifera solaris* showed that the highest conversion rate was achieved by pre-culturing for 48 h [37]. The above results indicated that the optimal pre-cultivation time varied with individual differences. Therefore, it is necessary to optimize the pre-culture time.

The orientation of the plant tissue explants placed on the medium is another important factor affecting the efficiency of plant transformation and regeneration [38]. The abaxial orientation of the leaf implies that the lower surface of the leaf is in contact with the medium, while the adaxial position means that the upper surface of the leaf is in contact with the medium [39]. In our pretest study, we found that more adventitious buds were produced during the regeneration stage when the abaxial side of the leaf was exposed to the medium than the adaxial side. Mazumdar et al. reported that the regeneration efficiency of the abaxial end of explants exposed to the medium was twice as high as that of the adaxial end [40]. Similarly, tomatoes of two different genotypes exhibited higher regeneration efficiency and higher shoot numbers per explant when placed abaxially than when exposed to medium in the adaxial direction [41], as confirmed in earlier studies [42,43].

The PEG/Mg^2+^ coating protocol in this study showed better stabilization of the transformation than the standard Spd/Ca^2+^ method. Due to the hygroscopicity, oxidizable nature, and deamination over time of spermidine (Spd) solutions, frozen aliquots should be replaced for fresh at least monthly [44]. Stock solutions of CaCl_2_ and Spd must be used separately, whereas PEG and MgCl_2_ can be prepared conveniently as a single stock solution that is stable for many years when stored at −20 °C. The PEG/Mg^2+^ procedure has been successfully applied to wheat for stable transformation [45].

The distance from the microcarrier to the target tissues can affect the velocity of the microparticles and thus the frequency of transformation [46]. As in previous studies, a target tissue distance of 9 cm was reported as the optimal propagation distance for bananas [47], wheat [48], and cumin [49]. We found that a target tissue distance of 9 cm was a significant factor in improving transformation frequency. This distance can reduce damage to a great extent and ensure the distribution of DNA microcarriers on target tissues [50].

Similar to Jähne et al., we found that changing the helium pressure in the range of 1100–1350 psi had no significant effect on the number of positive shoots [51]. In contrast to the results of this study, Jähne et al. found that lower (450–900 psi) or higher (2000–2200 psi) pressures reduced the frequency of transformation. When Harwood et al. used barley microspores pre-incubated for 1–4 days, they found that a lower pressure of 450 psi increased the number of GUS-positive microspores [52]. We also found that lower bombardment pressures increased the number of positive shoots. When Mentewab et al. compared the effect of using 650 psi or 1100 psi pressure, transient expression of microspores in culture for 1 day was observed only after using low pressure, while multicellular structures of 8 days were only observed when high pressure was applied [53]. It was demonstrated that the optimal helium burst pressure may depend on several factors related to the cell wall properties and the damaging effect of the treatment.

Comparing the number of surviving shoots, an overall optimal parameter was observed, i.e., precipitant agents MgCl_2_ + PEG, target tissue distance 9 cm, helium pressure 650 psi, bombardment once, plasmid DNA concentration 1.0 μg·μL^−1^, and chamber vacuum pressure 27 mmHg. In this study, the frequency of transformation obtained by biolistic transformation was 36.7%. The transformation efficiency of *E. breviscapus* was not reported for either *Agrobacterium*-mediated or particle bombardment transformation (three adventitious shoots were obtained). The higher transformation efficiency of the particle gun bombardment may be due to the attempted use of the hybrid orthogonal methods. Optimization strategies are highly efficient compared to the previous single-variable method, thus potentially facilitating genetic modification for improved traits.

## 4. Materials and Methods

### 4.1. Plant Material and Culture Conditions

Seeds of *E. breviscapus* were manually rubbed to remove their pappus, and the plump and undamaged seeds were selected for the experiment. Before inoculation, seeds were soaked for 4 h, dried for 3 h, and then sterilized. Under sterile conditions, seeds were soaked in 75% (*v*/*v*) ethanol for 8~10 s and washed 3~4 times with sterile water. After that, they were sterilized with 2.5% (*v*/*v*) NaClO for 8~10 min and rinsed with sterile water. Seeds were inoculated on Murashige and Skoog (MS) medium [54] hormone-free medium and cultured at 25 °C, 16 h/d light conditions of 4000 lx to obtain sterile seedlings.

### 4.2. Optimization of Explant Pre-Culture Time

Ten leaf explants were placed on each plate containing callus induction medium (CIM; MS + 1.0 mg·L^−1^ 6-BA + 0.1 mg·L^−1^ NAA + 3% sucrose + 0.4% phytagel, pH 5.8). A total of six conditions of 2 d, 3 d, 5 d, 7 d, 14 d, and 21 d were established in order to confirm the most suitable pre-culture time and the explants were incubated in the dark at 25 °C. After 4 h of osmotic treatment, the leaf explants were bombarded with the following parameters: 6 cm target distance, 27 mmHg, with 1100 psi rupture disks. The optimal pre-incubation time was evaluated by the frequency of transformed shoots. The methods of bombardment are described in 4.4 and 4.5.

### 4.3. Selection Pressure of Leaf Explants to Hygromycin B

The concentrations of Hyg were set at five levels of 0, 2.5, 5.0, 7.5, and 10.0 mg·L^−1^, and the medium was CIM. Leaf explants were placed abaxially and inoculated with each treatment and replicated three times. All the explants were cultured in light at 25 °C for 4 weeks and sub-cultured every 2 weeks. The critical concentration of Hyg was confirmed by counting the induction rate of resistant callus.

### 4.4. Preparation of Gold Microprojectile

Plasmid PBI-1300 (provided by Pro. Meng Wang, Institute of Genetics and Developmental Biology, Chinese Academy of Sciences) was isolated using the plasmid maxi kit (Omega, United States) according to the manufacturer’s protocol. Transformation conditions were confirmed by plasmid PBI-1300, which harbors the *eGFP* reporter gene and the *hpt* selectable gene, both driven by the cauliflower mosaic virus (CaMV) 35S promoter.

The gold particles must be sterilized before being used for DNA coating. An amount of 30 mg of 1.0 μm gold particles were added with 1 mL of 100% ice-cold ethanol and sonicated for 15 s, and were centrifuged at 3000 rpm for 60 s. The supernatant was carefully discarded and the gold particles were resuspended with 1 mL ice-cold ddH_2_O. The gold particles were centrifuged at 3000 rpm for 60 s and supernatant was removed. The washing step of ddH_2_O was repeated twice. Finally, the gold particles were suspended with 500 µL of 50% (*v*/*v*) sterile glycerin with a final concentration of 60 mg·mL^−1^. The above microcarriers were stored at −20 °C before use.

The DNA/gold-coating method consists of two levels of optimization (Table 1). The first method is based on Bio-Rad protocol. An amount of 5 μL DNA (1 μg·μL^−1^), 50 μL CaCl_2_ (2.5 M), and 20μL Spd (0.1 M) were added to 50 uL microparticle solution, and the mixture was vortexed for 2~3 min and left to stand for 1 min. The supernatant was discarded after centrifugation. The particles were washed with 140 μL of 70% ethanol and absolute ethanol. Finally, the pelleted DNA was suspended with 48 μL of absolute ethanol. The second method corresponded to the previous method [45]. An amount of 50 μL of gold particles was coated with 10 μL of DNA (1 μg·μL^−1^) and supplemented with 10 µL of PM solution (42% PEG 2000 and 560 mM MgCl_2_) under vortexing. The mixture was vortexed for 1 min and incubated for 20 min at room temperature. The suspension was centrifuged for 1~5 min. Then, the pelleted DNA was washed with 100 µL of 100% ethanol and was resuspended in 60 µL of 100% ethanol.

### 4.5. Microprojectile Bombardment

The bombardments were performed with a particle gun (PDS 1000/He, Bio-Rad). The transformation conditions of the particle gun were optimized using one 2-level factor (precipitation agents) and five 3-level factors (target tissue distance, helium pressure, bombardment times, plasmid DNA concentration, and chamber vacuum pressure). These parameters and the levels of the variables studied are shown in Table 3. A hybrid orthogonal table L_18_(2^1^ × 3^5^) was designed according to the above six parameters and different levels of each parameter [55], as shown in Table 6, where A to F represent precipitation agents, target tissue distance, helium pressure, number of bombardments, plasmid DNA concentration, and chamber vacuum pressure. Each factor was repeated three times, and the whole set of experiments was repeated twice.

### 4.6. Selection and Regeneration of Transformed Plants

The bombarded leaf explants were incubated in the dark at 25 °C for 10~16 h and then transferred to CIM. After 2 days, the transformed leaves were observed for expression of *eGFP* using Laser Scanning Confocal Microscope (LSM880NLO, Zeiss, German) under an excitation wavelength of 488 nm. The explants were then cut into small pieces of about 1 cm^2^, placed abaxially on fresh CIM, and cultured at 25 °C, 16 h/d light. After 2 weeks, the transformed cells were observed for the expression of *eGFP* through LSCM under an excitation wavelength of 488 nm. The explants were then placed abaxially on CIM containing 2.5 mg·L^−1^ Hyg. After three rounds of selection, the calluses were split into 3~5 pieces and transferred to the regeneration medium (RM; MS + 2.0 mg·L^−1^ 6-BA + 0.2 mg·L^−1^ NAA + 3% sucrose + 0.4% phytagel, pH 5.8) and incubated at 25 °C under 16 h/d light conditions of 4000 lx. After 2 weeks, green shoots were transferred to shoot elongation medium (SEM; 1/2MS + 2.0 mg·L^−1^ 6-BA + 0.2 mg·L^−1^ NAA + 3% sucrose + 0.4% phytagel, pH 5.8). For rooting formation, adventitious buds were transferred to root induction medium (RIM; 1/2MS + 0.3 mg·L^−1^ NAA + 0.5 mg·L^−1^ IBA + 3% sucrose + 0.4% phytagel, pH 5.8).

### 4.7. PCR Analysis

The genomic DNA of transformed plants was isolated using CTAB method [56]. PCR analysis was performed with *hpt* (selection maker gene)-specific primers (F: 5′-ATGAAAAAGCCTGAACTCACCG-3′; R: 5′-CTATTTCTTTGCCCTCGGACG-3′) to confirm the transformed strain. The denaturation temperatures used were 98 °C for 30 s, then 35 cycles for amplification with denaturation at 98 °C for 10 s, annealing at 60 °C for 30 s, extension at 72 °C for 20 s, and final extension at 72 °C for 7 min. Then, all the PCR products were sequenced by Sanger sequencing.

### 4.8. Statistical Analysis

The optimization experiment was repeated three times. The different levels of each experimental factor and the contribution rate of each factor were determined by range analysis and variance analysis. The statistical analysis of all data was calculated by formula. In the range analysis, the values of R and means of Ki were calculated:

$$R=\max\;(\bar{K_{i}})-\min\;(\bar{K_{i}})$$

The range analysis of the hybrid orthogonal experiment was used to adjust the range R value and compared with the adjusted R’ value, where r is the number of replicates for each level of parameters, and d is the conversion coefficient, which is related to the parameter level:

$$R^{\prime }=\mathrm{dR}\sqrt{r}$$

In the analysis of variance, the sum of squared deviations of the factors was calculated as follows:

$$SSj=\frac{1}{r}\overset{m}{\underset{i=1}{\sum }}K_{ij}^{2}-\frac{(\sum_{i=1}^{n}Xi)2}{n}\left(j=1,2,\ldots ,k\right)$$

In the analysis of variance, if the level of the factor is 2, the sum of squared deviations of the factors was calculated as follows:

$$\mathrm{SSj}=\frac{1}{n}(K_{1j}-K_{2j}{)}^{2}\;(j=1,2,\;...,\;k)$$

The significance of difference was calculated by *F*-test (* *p* < 0.05; ** *p* < 0.01).

## Figures and Tables

**Table 1:** Effects of Hyg on callus induction from leaf explants.

Antibiotic	Concentration (mg·L^−1^)	Callus Induction Rate (%)
Hyg	0.0	88.61 ± 5.11 *
	2.5	43.34 ± 3.67
	5.0	15.93 ± 4.90
	7.5	0
	10.0	0

* Values represent the mean (±SE) of three independent experiments.

**Table 2:** Effects of pre-culture day on transformation.

Pre-Culture Day (d)	Transformed Callus Frequency (%)
2	0
3	0
5	0
7	54.40 ± 17.40 *
14	59.67 ± 12.04
21	0

* Values represent the mean (±SE) of three independent experiments.

**Table 3:** Parameters and levels involved in this study.

Symbol	Parameters	Levels		
		1	2	3
A	Precipitation agents	CaCl_2_ + Spd	MgCl_2_ + PEG	-
B	Target tissue distance (cm)	3	6	9
C	Helium pressure (psi)	650	1350	1100
D	Number of bombardments	1	2	3
E	Plasmid DNA concentration (μg·μL^−1^)	1	1.5	2
F	Chamber vacuum pressure (mmHg)	26	27	28

**Table 4:** Range analysis of different parameters.

Parameters	K1 ^1^	K2 ^2^	K3 ^3^	R ^4^	R’ ^5^
Precipitation agents	62	96	-	34	59.13
Target tissue distance (cm)	50	24	84	60	54.04
Helium pressure (psi)	67	47	44	23	20.72
Number of bombardments	58	37	63	26	23.42
Plasmid DNA concentration (μg·μL^−1^)	72	39	47	33	29.72
Chamber vacuum pressure (mmHg)	42	60	56	18	16.21

^1^ K1, average of all values at level 1; ^2^ K2, average of all values at level 2; ^3^ K3, average of all values at level 3; ^4^ R, the range value of each level; ^5^ R’, adjust the range R and compare it with the adjusted R’.

**Table 5:** Variance analysis of different parameters.

Parameters	df ^a^	SS ^b^	MS ^c^	*F* ^d^	*p*
Precipitation agents	1	64.22	64.22	9.53	0.021 *
Target tissue distance (cm)	2	301.78	150.89	22.38	0.002 **
Helium pressure (psi)	2	52.11	26.06	3.87	0.083
Number of bombardments	2	63.44	31.72	4.71	0.059
Plasmid DNA concentration (μg·μL^−1^)	2	98.78	49.39	7.33	0.025 *
Chamber vacuum pressure (mmHg)	2	29.78	14.89	2.21	0.191
Error	6	40.44	6.74		
Total	17	650.56	38.27		

^a^ *df*, Degree of Freedom; ^b^ SS, Sums of Squares; ^c^ MS, Adjusted Mean Sums of Squares; ^d^ *F*, *F*-test statistic. * 0.01 ≤ *p* < 0.05, ** *p* < 0.01.

**Table 6:** L_18_(2^1^ × 3^5^) hybrid orthogonal table.

Group	A	B	C	D	E	F	Vacant Column
Group 1	1	1	1	1	1	1	1
Group 2	1	1	2	2	2	2	2
Group 3	1	1	3	3	3	3	3
Group 4	1	2	1	1	2	2	3
Group 5	1	2	2	2	3	3	1
Group 6	1	2	3	3	1	1	2
Group 7	1	3	1	2	1	3	2
Group 8	1	3	2	3	2	1	3
Group 9	1	3	3	1	3	2	1
Group 10	2	1	1	3	3	2	2
Group 11	2	1	2	1	1	3	3
Group 12	2	1	3	2	2	1	1
Group 13	2	2	1	2	3	1	3
Group 14	2	2	2	3	1	2	1
Group 15	2	2	3	1	2	3	2
Group 16	2	3	1	3	2	3	1
Group 17	2	3	2	1	3	1	2
Group 18	2	3	3	2	1	2	3

## Data Availability

Not applicable.

## Abbreviations

MS	Murashige and Skoog
LSCM	Laser Scanning Confocal Microscope
6-BA	6-Benzylaminopurine
NAA	1-Naphthylacetic acid
IBA	Indole-3-butyric acid
Hyg	Hygromycin B
CIM	Callus induction medium
RM	Regeneration medium
SEM	Shoot elongation medium
RIM	Root induction medium
CaMV	Cauliflower mosaic virus

## References

[1] Dong X., Qu S. (2022). Erigeron breviscapus (Vant.) Hand-Mazz.: A Promising Natural Neuroprotective Agent for Alzheimer’s Disease. Front. Pharmacol..

[2] Fan H., Lin P., Kang Q., Zhao Z.L., Wang J., Cheng J.Y. (2021). Metabolism and Pharmacological Mechanisms of Active Ingredients in Erigeron breviscapus. Curr. Drug Metab..

[3] Jiang L., Hu Y., He X., Lv Q., Wang T.H., Xia Q.J. (2017). Breviscapine reduces neuronal injury caused by traumatic brain injury insult: Partly associated with suppression of interleukin-6 expression. Neural Regen. Res..

[4] Deng M., Sun J., Peng L., Huang Y., Jiang W., Wu S., Zhou L., Chung S.K., Cheng X. (2022). Scutellarin acts on the AR-NOX axis to remediate oxidative stress injury in a mouse model of cerebral ischemia/reperfusion injury. Phytomedicine.

[5] Pengyue Z., Tao G., Hongyun H., Liqiang Y., Yihao D. (2017). Breviscapine confers a neuroprotective efficacy against transient focal cerebral ischemia by attenuating neuronal and astrocytic autophagy in the penumbra. Biomed. Pharmacother..

[6] Wang L., Ma Q. (2018). Clinical benefits and pharmacology of scutellarin: A comprehensive review. Pharmacol. Ther..

[7] Wang J., Tan J., Luo J., Huang P., Zhou W., Chen L., Long L., Zhang L.M., Zhu B., Yang L. (2017). Enhancement of scutellarin oral delivery efficacy by vitamin B12-modified amphiphilic chitosan derivatives to treat type II diabetes induced-retinopathy. J. Nanobiotechnol..

[8] Tang G., Li S., Zhang C., Chen H., Wang N., Feng Y. (2021). Clinical efficacies, underlying mechanisms and molecular targets of Chinese medicines for diabetic nephropathy treatment and management. Acta Pharm Sin B..

[9] Safdari M.R., Shakeri F., Mohammadi A., Bibak B., Alesheikh P., Jamialahmadi T., Sathyapalan T., Sahebkar A. (2021). Role of Herbal Medicines in the Management of Brain Injury. Adv. Exp. Med. Biol..

[10] Avagimyan A., Gvianishvili T., Gogiashvili L., Kakturskiy L., Sarrafzadegan N., Aznauryan A. (2023). Chemotherapy, hypothyroidism and oral dysbiosis as a novel risk factor of cardiovascular pathology development. Curr. Probl. Cardiol..

[11] Liu X., Cheng J., Zhang G., Ding W., Duan L., Yang J., Kui L., Cheng X., Ruan J., Fan W. (2018). Engineering yeast for the production of breviscapine by genomic analysis and synthetic biology approaches. Nat. Commun..

[12] Wang W., Xu J., Fang H., Li Z., Li M. (2020). Advances and challenges in medicinal plant breeding. Plant Sci..

[13] Hwang H.-H., Yu M., Lai E.-M. (2017). Agrobacterium-mediated plant transformation: Biology and applications. Arab. Book.

[14] Chen Z., Debernardi J.M., Dubcovsky J., Gallavotti A. (2022). Recent advances in crop transformation technologies. Nat. Plants.

[15] Mohammed S., Abd Samad A., Rahmat Z. (2019). Agrobacterium-mediated transformation of rice: Constraints and possible solutions. Rice Sci..

[16] Ahmed R.I., Ding A., Xie M., Kong Y. (2018). Progress in Optimization of Agrobacterium-Mediated Transformation in Sorghum (Sorghum bicolor). Int. J. Mol. Sci..

[17] Lacroix B., Citovsky V. (2020). Biolistic approach for transient gene expression studies in plants. Biolistic DNA Delivery in Plants: Methods.

[18] Chandrasekaran R., Rajiv P., Abd-Elsalam K.A. (2020). Carbon nanotubes: Plant gene delivery and genome editing. Carbon Nanomaterials for Agri-Food and Environmental Applications.

[19] Sood P., Bhattacharya A., Sood A. (2011). Problems and possibilities of monocot transformation. Biol. Plant..

[20] Van Eck J. (2018). The Status of Setaria viridis Transformation: Agrobacterium-Mediated to Floral Dip. Front. Plant Sci..

[21] Altpeter F., Baisakh N., Beachy R., Bock R., Capell T., Christou P., Daniell H., Datta K., Datta S., Dix P.J. (2005). Particle bombardment and the genetic enhancement of crops: Myths and realities. Mol. Breed..

[22] Ahmar S., Mahmood T., Fiaz S., Mora-Poblete F., Shafique M.S., Chattha M.S., Jung K.H. (2021). Advantage of Nanotechnology-Based Genome Editing System and Its Application in Crop Improvement. Front. Plant Sci..

[23] Basso M.F., Arraes F.B.M., Grossi-de-Sa M., Moreira V.J.V., Alves-Ferreira M., Grossi-de-Sa M.F. (2020). Insights Into Genetic and Molecular Elements for Transgenic Crop Development. Front. Plant Sci..

[24] Liu C.Z., Gao M., Guo B. (2008). Plant regeneration of Erigeron breviscapus (vant.) Hand. Mazz. and its chromatographic fingerprint analysis for quality control. Plant Cell Rep..

[25] Zhang L., Liu C., Lin L., Chen W. (2007). Callus Induction and Adventitious Shoot Regeneration from Petiole of Erigeron breviscapus. Plant Prod. Sci..

[26] Zhang Z., Zhao Z., Yang W., Zhang J., Yang M., Jin H. (2009). Preliminary study on anther culture of Erigeron breviscapus. Bull. Bot. Res..

[27] He S., Dong X., Zhang G., Fan W., Duan S., Shi H., Li D., Li R., Chen G., Long G. (2021). High quality genome of Erigeron breviscapus provides a reference for herbal plants in Asteraceae. Mol. Ecol. Resour..

[28] Qiu L. (1994). Genetic transformation of Erigeron breviscapus induced by particle gun with a few affecting factors. Chin. Tradit. Herb. Drugs.

[29] Wang Z.-Y., Ge Y. (2006). Recent advances in genetic transformation of forage and turf grasses. Vitr. Cell. Dev. Biol.-Plant.

[30] Zárate R., Verpoorte R. (2007). Strategies for the genetic modification of the medicinal plant *Catharanthus roseus* (L.) G. Don. Phytochem. Rev..

[31] Franklin G., Oliveira M., Dias A.C.P. (2007). Production of transgenic Hypericum perforatum plants via particle bombardment-mediated transformation of novel organogenic cell suspension cultures. Plant Sci..

[32] Lai K.-S., Abdullah P., Yusoff K., Mahmood M. (2011). An efficient protocol for particle bombardment-mediated transformation of Centella asiatica callus. Acta Physiol. Plant..

[33] Zhao Y., Zhang Y., Su P., Yang J., Huang L., Gao W. (2018). Genetic transformation system for woody plant Tripterygium wilfordii and its application to product natural celastrol. Front. Plant Sci..

[34] Narra M., Ellendula R., Kota S., Kalva B., Velivela Y., Abbagani S. (2018). Efficient genetic transformation of Momordica charantia L. by microprojectile bombardment. 3 Biotech.

[35] Srinivas K., Muralikrishna N., Kumar K.B., Raghu E., Mahender A., Kiranmayee K., Yashodahara V., Sadanandam A. (2016). Biolistic transformation of *Scoparia dulcis* L.. Physiol. Mol. Biol. Plants.

[36] Folling L., Olesen A. (2001). Transformation of wheat (*Triticum aestivum* L.) microspore-derived callus and microspores by particle bombardment. Plant Cell Rep..

[37] Naser I., Yabu Y., Maeda Y., Tanaka T. (2022). Highly Efficient Genetic Transformation Methods for the Marine Oleaginous Diatom Fistulifera solaris. Mar. Biotechnol..

[38] Bhatia P., Ashwath N., Midmore D.J. (2005). Effects of genotype, explant orientation, and wounding on shoot regeneration in tomato. Vitr. Cell. Dev. Biol. Plant.

[39] George E.F. (1993). Plant Propagation by Tissue Culture.

[40] Mazumdar P., Basu A., Paul A., Mahanta C., Sahoo L. (2010). Age and orientation of the cotyledonary leaf explants determine the efficiency of de novo plant regeneration and Agrobacterium tumefaciens-mediated transformation in *Jatropha curcas* L.. South Afr. J. Bot..

[41] Rani T., Yadav R.C., Yadav N.R., Kumar M. (2013). Effect of explant orientation on shoot regeneration in tomato (*Lycopersicon esculentum*). Indian J. Agric. Sci..

[42] Welander M., Maheswaran G. (1992). Shoot regeneration from leaf explants of dwarfing apple rootstocks. J. Plant Physiol..

[43] Bartish I., Korkhovoi V. (1997). The composition of nutrient medium and the efficiency of shoot induction in vitro from apple leaf explants. Russ. J. Plant Physiol..

[44] Sanford J.C., Smith F.D., Russell J.A. (1993). Optimizing the biolistic process for different biological applications. Method Enzymol..

[45] Ismagul A., Yang N., Maltseva E., Iskakova G., Mazonka I., Skiba Y., Bi H., Eliby S., Jatayev S., Shavrukov Y. (2018). A biolistic method for high-throughput production of transgenic wheat plants with single gene insertions. BMC Plant Biol..

[46] Petrillo C.P., Carneiro N.P., Purcino A.Á.C., Carvalho C.H.S., Alves J.D., Carneiro A.A. (2008). Optimization of particle bombardment parameters for the genetic transformation of Brazilian maize inbred lines. Pesqui. Agropecuária Bras..

[47] Mahdavi F., Mahmood M., Noor N.M. (2014). Optimization of particle bombardment parameters for DNA delivery into the male flowers of banana. Biologia.

[48] Gharanjik S., Moieni A., Mousavi A., Alizadeh H. (2008). Optimization of transient expression of uidA gene in androgenic embryos of wheat (*Triticum aestivum* L. cv. Falat) via particle bombardment. Iran. J. Biotechnol..

[49] Singh N., Mishra A., Joshi M., Jha B. (2010). Microprojectile bombardment mediated genetic transformation of embryo axes and plant regeneration in cumin (*Cuminum cyminum* L.). Plant Cell Tissue Organ Cult..

[50] Vasudevan R.A., Nadimuthu K., Ramachandran S. (2013). Method of High Frequency Regeneration of Sorghum. U.S. Patent.

[51] Jähne A., Becker D., Brettschneider R., Lörz H. (1994). Regeneration of transgenic, microspore-derived, fertile barley. Theor. Appl. Genet..

[52] Harwood W.A., Bean S.J., Chen D.F., Mullineaux P.M., Snape J.W. (1995). Transformation studies in Hordeum vulgare using a highly regenerable microspore system. Euphytica.

[53] Mentewab A., Letellier V., Marque C., Sarrafi A. (1999). Use of anthocyanin biosynthesis stimulatory genes as markers for the genetic transformation of haploid embryos and isolated microspores in wheat. Cereal Res. Commun..

[54] Classic Murashige T., Skoog F. (1962). A revised medium for rapid growth and bioassays with tobacco tissue cultures. Physiol. Plant.

[55] Aguilar-Zarate P., Cruz-Hernandez M.A., Montañez J.C., Belmares-Cerda R.E., Aguilar C.N. (2014). Enhancement of tannase production by Lactobacillus plantarum CIR1: Validation in gas-lift bioreactor. Bioprocess Biosyst. Eng..

[56] Murray M.G., Thompson W.F. (1980). Rapid isolation of high molecular weight plant DNA. Nucleic Acids Res..

